# Neither Replication nor Simulation Supports a Role for the Axon Guidance Pathway in the Genetics of Parkinson's Disease

**DOI:** 10.1371/journal.pone.0002707

**Published:** 2008-07-16

**Authors:** Yonghong Li, Charles Rowland, Georgia Xiromerisiou, Robert J. Lagier, Steven J. Schrodi, Efthimios Dradiotis, David Ross, Nam Bui, Joseph Catanese, Konstantinos Aggelakis, Andrew Grupe, Georgios Hadjigeorgiou

**Affiliations:** 1 Celera, Alameda, California, United States of America; 2 Department of Neurology, University of Thessaly Medical School, Larissa, Greece; 3 Institute of Biomedical Research & Technology, CERETETH, Larissa, Greece; Vrije Universiteit Medical Centre, Netherlands

## Abstract

Susceptibility to sporadic Parkinson's disease (PD) is thought to be influenced by both genetic and environmental factors and their interaction with each other. Statistical models including multiple variants in axon guidance pathway genes have recently been purported to be capable of predicting PD risk, survival free of the disease and age at disease onset; however the specific models have not undergone independent validation. Here we tested the best proposed risk panel of 23 single nucleotide polymorphisms (SNPs) in two PD sample sets, with a total of 525 cases and 518 controls. By single marker analysis, only one marker was significantly associated with PD risk in one of our sample sets (rs6692804: *P* = 0.03). Multi-marker analysis using the reported model found a mild association in one sample set (two sided *P* = 0.049, odds ratio for each score change = 1.07) but no significance in the other (two sided *P* = 0.98, odds ratio = 1), a stark contrast to the reported strong association with PD risk (*P* = 4.64×10^−38^, odds ratio as high as 90.8). Following a procedure similar to that used to build the reported model, simulated multi-marker models containing SNPs from randomly chosen genes in a genome wide PD dataset produced *P*-values that were highly significant and indistinguishable from similar models where disease status was permuted (3.13×10^−23^ to 4.90×10^−64^), demonstrating the potential for overfitting in the model building process. Together, these results challenge the robustness of the reported panel of genetic markers to predict PD risk in particular and a role of the axon guidance pathway in PD genetics in general.

## Introduction

Parkinson's disease (PD) is the second most prevalent neurodegenerative disease only after Alzheimer's disease. The majority of cases are sporadic and usually manifest symptoms at 50 years or older while a small proportion of cases are inherited in Mendelian fashion. While several known mutations lead to the Mendelian forms of PD (for a recent review, see [1]), much remains to be uncovered to understand genetic causes of sporadic PD. A single variant in the leucine-rich repeat kinase 2 (*LRRK2*), Gly2019Ser, may explain ∼2% of the sporadic cases [2] and almost as many as 30% of cases in Ashkenazi Jews and North African Arab populations [3], [4]. A dinucleotide repeat sequence polymorphism in the promoter region of the α-synuclein gene (*SCNA*) and a haplotype in the microtubule-associated protein tau gene (*MAPT*) are associated with increased PD risk (for recent meta-analyses of large sample sets, see [5], [6]). Association of various other polymorphisms with PD risk has also been reported (www.pdgene.org), but requires further validation before more definitive conclusion can be made as to whether any are genuine PD risk factors.

Two recent genome-wide association (GWA) studies have examined about half a million single nucleotide polymorphisms (SNPs) for their association with PD [7], [8]. Although a number of putative disease markers have been proposed, independent replication studies have not confirmed any novel, significant findings [9]–[14]. Non-replication may be due to false positive findings in the initial study, which can be exacerbated by massive multiple testing performed in GWA studies, or various other possible factors, such as insufficient power, contributed by modest effect sizes, small sample collections, or genetic heterogeneity [15]. Furthermore, the initial GWA studies only reported results for single marker analyses and had not investigated interactions of multiple genetic risk factors that are thought to underpin common complex diseases such as PD.

Based on the hypothesis that the joint actions of common gene variants within certain pathways may play a major role in predisposing to complex diseases, Lesnick and colleagues recently reported that a combination of SNPs in axon-guidance pathway genes is a strong predictor of susceptibility to PD, survival free of the disease, or age at disease onset [16]. After mining a genome-wide SNP dataset with 443 case control pairs, they identified 1,460 markers in 128 axon-guidance pathway genes. They built several models, each with 20∼40 of the identified markers, that show significant association with PD (e.g. odds ratios [OR] of 4.6, 15.4 and 90.8 for the second, third and fourth quartiles of their predicted probability of PD as compared with the first quartile and *P* = 4.64×10^−38^ for PD susceptibility). Subsequently, they analyzed another genome-wide SNP dataset and constructed similar models with largely different SNPs. They observed significant association of these models with PD as well. Remarkably in a separate study, they further reported that models of axon guidance pathway gene variants were also strong predictors of another neurodegenerative disease, amyotrophic lateral sclerosis (ALS), with extraordinary statistical evidence (e.g. OR = 1739.73 for the fourth as compared with the first quartile of predicted probability of ALS, *P* = 2.92×10^−60^ for susceptibility to ALS) [17]. While a causal connection between the axon guidance pathway and PD is appealing and the observed significance appears to be exceedingly strong, this finding remains to be validated for the same markers and model in an independent sample set. Although the Lesnick *et al.* replication model uses SNPs from the same genes as their initial models, the replication SNPs differ from the original set of SNPs, thus a *bona fide* replication is lacking.

Here we chose to validate Lesnick *et al*'s initial model that was a strong predictor of PD susceptibility by testing the set of 23 SNPs from the original report in two independent PD case control sample sets, with a total of 525 cases and 518 controls (Table 1). We individually genotyped these markers and then tested the proposed model for its ability to predict risk to the disease. The findings and its implication on the method of pathway association studies are presented.

**Table 1:** Sample set characteristics.

	Celera Sample Set		Thessaly Sample Set	
	Cases	Controls	Cases	Controls
*No.* Subjects	311	311	214	207
% Female	53.1	53.1	52.3	50.7
AAO (±SD)*	63.8 (8.9)	n/a	64.2 (9.8)	n/a
AAO Range	50–87	n/a	32–87	n/a
AAS (±SD)**	70.1 (8.5)	70.2 (8.5)	69.5 (9.7)	60.0 (16.8)
AAS Range	52–90	52–90	32–93	18–89

* AAO: age at onset in years.

** AAS: age at sampling in years.

## Results

In their hypothesis generation sample set, Lesnick and colleagues reported the identification of a PD risk signature composed of 23 SNPs in the axon guidance pathway [16]. We individually genotyped these 23 SNPs in two PD case control sample sets (Table 1), as described in the Materials and Methods. Except for one marker in the Celera cases (rs17468382: *P_HWE_* = 0.0052) and another in the Celera controls (rs17641276: *P_HWE_* = 0.012), none of the other markers significantly deviated from Hardy-Weinberg Equilibrium (HWE) in their genotype distribution (All *P_HWE_*>0.05). As these were relatively mild deviations from HWE and since we could not discern any obvious genotype errors, we decided to keep these two markers for further analysis. Allelic association tests identified one marker, rs6692804, in *GNAI3* on chromosome 1 showing significance in the Thessaly sample set (*P* = 0.034). Allele frequencies of this marker in the Celera cases and controls were, however, in the opposite direction to the Thessaly sample set, rendering the marker non-significant in a meta-analysis of the combined sample sets (Mantel-Haenszel *P_combined_* = 0.88, Breslow-Day OR homogeneity *P* = 0.0075).

**Figure 1:**
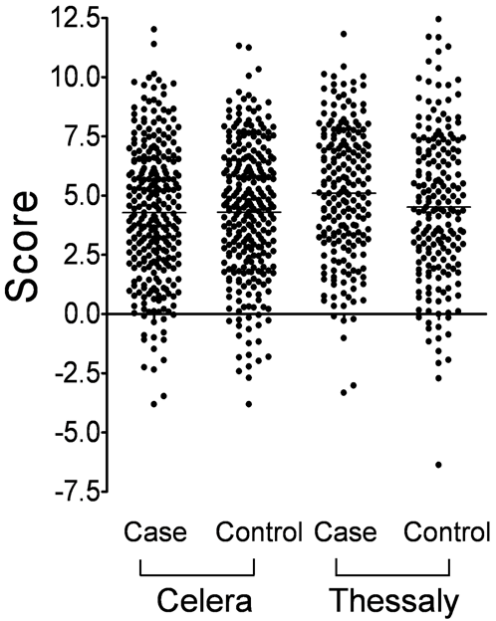
Dot plot showing regression scores of individual cases and controls in the Celera and Thessaly sample sets. A score was calculated for each individual as the sum of the specified main and interaction effects shown in Table 2.

We then coded each marker individually according to the genetic model reported in the original paper, ie, additive, dominant or recessive as presented in Table 2 of the Lesnick *et al.* study and examined whether the model reported by Lesnick *et al.* would be associated with PD susceptibility in the two sample sets we tested. A regression score was calculated for each subject based on the coefficients given in the Lesnick *et al.* publication (Figure 1), followed by testing for association with disease status. No significant association was observed in the Celera sample set at the 0.05 level, but there was a borderline significance in the Thessaly sample set (Table 2). Thus, in the latter sample set, there was an estimated 7% increase (95% CI: 0% to 14%) in the odds of PD for each one unit increase in the score, and this effect is in the same direction as reported by Lesnick *et al.* (Table 2). We then divided the subjects into quartiles according to their score and found the odds ratios (95% CIs) for the 2^nd^, 3^rd^ and 4^th^ quartiles as compared to the lowest quartile were 1.44 (0.82 to 2.51), 1.59 (0.91 to 2.77), and 1.83 (1.05 to 3.21) respectively (Table 3).

**Table 2:** Association results for the putative axon guidance pathway model with Parkinson's risk.

Sample set	OR (95% CI)	*P*-value
Celera	1.0 (0.94–1.06)	0.98
Thessaly	1.07 (1.0–1.14)	0.049

note: A score for each subject was calculated based on the coefficients given in the Lesnick paper.

Odds ratio represents the increase for each one unit change in the score.

**Table 3:** Odds ratio analysis for the groups defined by predicted PD probability in the Thessaly sample set.

Quartile of Predicted Probability of PD*	Odds Ratio Estimate	95% Wald Confidence Limits		*P*-overall model**
		Lower	Upper	
1 (<0.476)	1	NA	NA	0.18
2 (.476–.510)	1.44	0.82	2.51	
3 (.510–.546)	1.59	0.91	2.77	
4 (>.546)	1.83	1.05	3.21	

* Because we didn't have data in the nominal groups that Lesnick et al. defined (our predicted probabilities ranged from 0.328 to 0.634), we did not use the Lesnick et al. groupings. To be able to calculate odds ratios we broke the predicted probabilities into quartiles and calculated the odds ratios for each of the groups.

** Wald test with 3 degrees of freedom.

Additional models in the Celera sample set did not show association between the 23 markers and PD risk. Specifically, a logistic regression model which included the 23 main effects and 10 interaction terms as specified by Lesnick *et al.* but allowed the coefficients to be estimated by the data in the Celera sample set, was not significant (Likelihood ratio test *P*-value for significance of the model = 0.87). By this analysis, only 1 marker, rs16830689, was significant in the Celera sample set (*P* = 0.049), and another, rs6692804, in the Thessaly sample set (*P* = 0.027) (Table 4). This contrasts with the Lesnick *et al.* dataset where most markers were highly significant and had very large odds ratios. In addition, we examined the reported significant SNP-SNP interaction terms (*P*<0.05) and found no significant interaction between any of the 10 SNP pairs and PD risk in either of our sample sets (all *P*>0.05; Table 5). A backward stepwise selection procedure beginning with these same 23 main effects and 10 interaction terms terminated with none of these terms meeting the criteria to remain in the model. Similar analyses with the Thessaly sample set yielded a non-significant model when the coefficients for the main and interaction effects were estimated from the data (Likelihood ratio test *P*-value for significance of the model = 0.26) while the backward stepwise selection procedure produced a final model with rs6492998 as the single term remaining in the model (OR = 0.56, 95% CI 0.34 to 0.93; *P* = 0.026).

**Table 4:** Single marker association with Parkinson's disease risk according to the models from the Lesnick *et al.* publication.

Gene Symbol	rs_Number	Model*	Celera Sample Set		Thessaly Sample Set		Lesnick et al.**	
			Odds Ratio (95% CI)	*P*-value	Odds Ratio (95% CI)	*P*-value	Odds Ratio (95% CI)	*P*-value
CDC42	rs12740705	DOM	0.84 (0.56–1.27)	0.43	1.45 (0.88–2.37)	0.14	0.16 (0.06–0.41)	1.3E-04
CHP	rs6492998	DOM	1.24 (0.68–2.26)	0.48	0.46 (0.19–1.10)	0.08	0.21 (0.09–0.47)	1.6E-04
DCC	rs17468382	DOM	0.91 (0.32–2.57)	0.86	0.47 (0.05–4.13)	0.50	0.07 (0.01–0.67)	2.1E-02
EFNA5	rs153690	DOM	1.33 (0.62–2.86)	0.46	1.73 (0.70–4.25)	0.23	0.80 (0.29–2.21)	6.7E-01
EPHA4	rs13386128	ADD	1.06 (0.62–1.80)	0.82	1.70 (0.91–3.17)	0.10	16.29 (5.95–44.59)	5.6E-08
EPHB1	rs2030737	DOM	0.87 (0.56–1.33)	0.53	0.79 (0.46–1.35)	0.40	2.60 (1.23–5.51)	1.2E-02
EPHB2	rs10917325	ADD	1.12 (0.64–1.96)	0.68	1.16 (0.55–2.44)	0.68	0.11(0.05–0.28)	1.6E-06
FYN	rs6910116	REC	2.22 (0.41–11.8)	0.35	9.86 (0.42–226.)	0.15	59.95 (5.01–717.33)	1.2E-03
GNAI3	rs6692804	REC	2.45 (0.72–8.29)	0.15	0.21 (0.05–0.83)	0.03	0.07 (0.01–0.32)	6.3E-04
GSK3B	rs16830689	REC	0.35 (0.12–0.99)	0.05	0.55 (0.19–1.55)	0.26	0.09 (0.02–0.44)	2.9E-03
MRAS	rs4678260	ADD	0.83 (0.57–1.19)	0.32	1.06 (0.63–1.78)	0.82	0.97 (0.57–1.65)	9.1E-01
NTNG1	rs11185076	REC	1.12 (0.16–7.57)	0.90	5.32 (0-Inf)	0.98	0.02 (0.001–0.32)	5.7E-03
PAK4	rs17641276	REC	1.38 (0.83–2.30)	0.21	1.15 (0.64–2.04)	0.63	9.55 (3.73–24.43)	2.5E-06
PAK7	rs2072952	ADD	1.31 (0.74–2.32)	0.35	1.14 (0.49–2.63)	0.75	1.70 (0.75–3.90)	2.1E-01
PLXNA2	rs6656034	ADD	0.92 (0.70–1.21)	0.58	0.95 (0.69–1.30)	0.78	2.92 (1.75–4.87)	4.0E-05
PLXNC1	rs2068435	ADD	1.03 (0.73–1.45)	0.86	1.11 (0.73–1.68)	0.62	0.51 (0.25–1.02)	5.5E-02
PPP3CA	rs2044041	DOM	0.73 (0.50–1.06)	0.10	1.03 (0.64–1.66)	0.89	5.72 (2.84–11.51)	1.1E-06
RAC2	rs739043	REC	0.72 (0.41–1.29)	0.28	0.81 (0.41–1.59)	0.55	0.16 (0.069–0.39)	4.8E-05
RRAS2	rs2970332	DOM	1.08 (0.47–2.48)	0.85	1.85 (0.56–6.10)	0.31	8.78 (2.45–31.44)	8.5E–05
SEMA5A	rs12658266	DOM	1.16 (0.75–1.79)	0.48	1.14 (0.69–1.90)	0.59	0.22 (0.10–0.49)	2.0E-04
SLIT3	rs9688032	DOM	1.00 (0.45–2.22)	0.98	0.70 (0.26–1.86)	0.49	17.46 (4.61–66.07)	2.5E-05
UNC5C	rs11097458	ADD	0.84 (0.64–1.11)	0.23	1.00 (0.73–1.36)	0.97	2.25 (1.41–3.61)	7.2E-04
UNC5C	rs4444836	DOM	1.15 (0.77–1.72)	0.47	1.10 (0.68–1.77)	0.69	0.38 (0.17–0.85)	1.8E-02

* ADD: log additive; DOM: Mendelian dominant; REC, Mendelian recessive.

** Lesnick et al. PLoS Genet 3(6): e98. Table 1.

**Table 5:** SNP-SNP interaction with Parkinson's disease risk according to the models from the Lesnick *et al.* publication.

Interaction		Celera Sample Set		Thessaly Sample Set		Lesnick et al.	
Gene-Gene	SNP-SNP	Odds Ratio (95% CI)	*P*_interaction_	Odds Ratio (95% CI)	*P*_interaction_	Odds Ratio (95% CI)	*P*_interaction_
DCC*PAK4	rs17468382*rs17641276	3.20 (0.46–21.9)	0.24	1.18 (0.03–37.6)	0.92	0.02 (0.002–0.19)	6.0E-04
EPHA4*FYN	rs13386128*rs6910116	0.78 (0.34–1.76)	0.55	0.66 (0.28–1.54)	0.35	0.16 (0.05–0.53)	2.8E-03
EPHA4*PAK7	rs13386128*rs2072952	0.96 (0.59–1.55)	0.88	0.62 (0.36–1.07)	0.09	0.26 (0.11–0.62)	2.7E-03
EPHB2*EFNA5	rs10917325*rs153690	0.90 (0.48–1.68)	0.76	0.66 (0.29–1.49)	0.33	5.25 (2.10–13.13)	4.0E-04
FYN*RRAS2	rs6910116*rs2970332	0.58 (0.10–3.24)	0.54	0.14 (0.00–3.14)	0.22	0.04 (0.003–0.50)	1.3E-02
FYN*SLIT3	rs6910116*rs9688032	0.80 (0.33–1.93)	0.63	0.84 (0.29–2.43)	0.76	4.52 (1.32–15.47)	1.6E-02
MRAS*SLIT3	rs4678260*rs9688032	1.02 (0.60–1.74)	0.92	1.07 (0.56–2.08)	0.82	0.18 (0.08–0.41)	5.9E-05
PAK7*CHP	rs2072952*rs6492998	0.75 (0.40–1.39)	0.37	1.40 (0.58–3.36)	0.45	2.94 (1.23–7.01)	1.5E-02
SEMA5A*RAC2	rs12658266* rs739043	1.01 (0.38–2.72)	0.97	0.50 (0.15–1.64)	0.26	4.95 (1.37–17.91)	1.5E-02
UNC5C*DCC	rs11097458*rs17468382	0.92 (0.37–2.27)	0.86	1.39 (0.32–5.90)	0.65	6.89 (1.20–39.51)	3.0E-02

**Figure 2:**
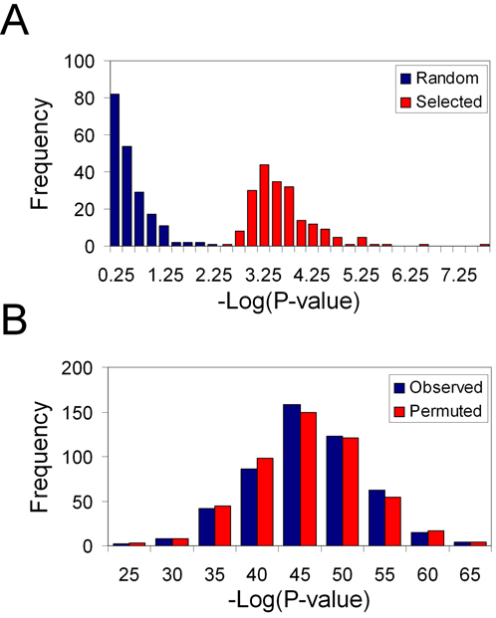
Simulation analysis. (A) Histogram of randomly-selected SNPs and highly-selected SNPs, both generated under a null model and uncorrected for multiple testing. The selected SNPs were obtained by taking the most significant SNP from a set of 1,400 null SNPs. The random SNPs were obtained by randomly-selecting a marker from the 198k SNPs. The 200 presented data points were generated from 200 iterations of the simulation. (B) Distribution of −log(*P*-value) for global likelihood ratio test from the observed method and the permuted method that mimic Lesnick's model selection scheme (see Materials and Methods). *P*-values were tallied from 500 observed models and 500 permuted models.

In their paper, Lesnick *et al.* compared the significance of their axon-guidance pathway SNP models to the significance of SNP models containing an equal number of markers that were randomly selected from throughout the genome. However, by comparing their observed results to randomly selected SNPs from the entire dataset (198k SNPs), they are generating a null distribution uncorrected for the multiple testing and selection of the most significant SNPs chosen from among ∼1,400 SNPs (the number of SNPs in the axon guidance pathway) in their dataset. To illustrate this further, we carried out a simple simulation where 1,400 SNPs were generated under a null model of independence between phenotype and genotypes and the most significant SNP was retained. This selected SNP was plotted alongside a SNP randomly selected from 198k SNPs generated under the same null model. This procedure was iterated 200 times and the results presented in a histogram of the −log_10_ *P*-value (association *P*-value uncorrected for multiple testing) (Figure 2A). There is a clear difference between the distribution of *P*-values from the highly-selected SNPs (from the 1,400 null SNPs) and the randomly-selected SNPs (from 198k null SNPs) even though they are all generated under the identical null model. Although this is an oversimplification of the procedure used by Lesnick *et al.*, it does serve to illustrate how overfitting can occur from results taken from a large number of tests if correction for multiple testing is not performed.

In order to generate a null distribution corrected for the overfitting caused by selection of a model from a large number of SNPs, we simulated the Lesnick *et al.* model selection process using the Fung *et al.* publicly available Parkinson data [8] (N = 540; 269 cases *vs* 271 controls). We iterated a model selection procedure very similar to the Lesnick *et al.* process (details in statistical methods) 500 times. For each iteration, two datasets were created from the Fung data: one where the disease status remained intact for each of the 540 study participants (Observed Method) and one where the disease status was permuted to randomize the association of PD among those individuals (Permuted Method). The selection procedure then generated a final model for each dataset. The number of individual SNPs achieving univariate significance (*P*<0.05) ranged from 82 to 174 for the 500 iterations of the observed model and from 83 to 174 for the permuted models. A stepwise logistic regression procedure with selection from the sets of significant SNPs resulted in final models where the number of SNPs included ranged from 14 to 58 for the observed models and from 15 to 57 for the permuted models. Histograms of the −log_10_ *P*-values for the final models for both selection methods are shown in Figure 2B. The distribution of *P*-values for SNP models from the observed method appears to be indistinguishable from those of the permuted method and the range of *P*-values (3.13×10^−23^ to 4.90×10^−64^) spans the *P*-value (3.93×10^−44^) from Lesnick's final model for PD susceptibility on this same dataset built from SNPs in the axon guidance pathway genes.

## Discussion

Findings from GWA studies suggest that individual genetic variants make very modest contributions to the risk of common complex diseases, such as type II diabetes and autoimmune diseases (see, for example, [18]–[20]). The small genetic effect is in part responsible for the often-reported false positive associations [21] and dictates replication of initial findings in large, often tens of thousands of case and control samples. In the case of GWA studies of PD, no novel genetic markers have been convincingly identified. The assumption that multiple disturbances in certain pathways lead to the manifestation of disease is appealing – for example, the amyloid β pathway has been linked to mutations that cause Mendelian forms of Alzheimer's disease [22] and mitochondrial dysfunction and ubiquitin-proteasomal pathway have been postulated to be key to PD pathogenesis [23], [24]. Lesnick *et al.* hypothesized that the axon guidance pathway is critically involved in PD risk and provided a multi-marker genetic model capable of strongly predicting PD risk (odds ratio of Q4 *vs* Q1 = 90.8, *P* = 4.64×10^−38^). Wang and colleagues analyzed the same dataset (Maraganore *et al.* dataset [7]) using a method analogous to the pathway clustering analysis for micro-arrays. Their analysis also identified axon guidance as the most significant pathway that showed an over-representation of disease-associated markers compared to the null hypothesis, although they did not provide any genetic model with specific markers [25]. However, the axon guidance pathway did not make the short list of the most significant pathways in a similar analysis of another PD dataset (Fung *et al.* dataset [8], which contains ∼400,000 SNPs compared to ∼198,000 SNPs in the Maraganore *et al.* dataset and is similar in sample size to the Maraganore *et al*'s replication sample set) [25]. There might be multiple reasons for these discrepant findings, including differences in choice of the genotyped SNPs between two datasets and the possibility of a false positive result in the Maraganore *et al.* dataset. Consistent with the Wang *et al.* results, we were unable to confirm the putative risk panel in the Celera sample set which contains overlapping samples with the Fung *et al.* dataset (both draw case control samples from the NINDS Human Genetics Resource). Furthermore, no significant association was observed after exploring additional genetic models with the 23 markers.

Although the association of the multi-marker panel with PD risk showed a borderline replication in the Thessaly sample set, the association was not nearly as strong as that observed in the replication sample set used by Lesnick *et al.* (ORs for 2^nd^, 3^rd^ and 4^th^ quartiles of predicted probability of PD as compared to lowest quartile of 1.44, 1.54 and 1.83 in the Thessaly sample set versus 7.86, 16.14 and 121.14 in Lesnick et al.) These results, together with the non-replication of the putative model in the Celera sample set, challenge the specific risk model proposed by Lesnick *et al.* and thus a genetic contribution of the axon guidance pathway to PD risk in general. Although Lesnick *et al.* presented “replication” data in their initial report, the methods used for replication employed by them and by our study are fundamentally different. We tested the same set of SNPs and genetic model identified in the hypothesis generation sample set, whereas Lesnick *et al.* used different SNPs in the same set of genes – a main limitation of their study due to the fact that not all markers typed in their hypothesis generation sample set had been typed in the replication sample set. In the replication by Lesnick *et al.*, SNPs were selected through a multi-stage process that involved the selection process in the hypothesis generating sample set. Such multi-stage selection procedures employed during both hypothesis generation and hypothesis validation stages are liable to generate overfit models (see further discussion later), whereas our replication of a single model (determined *a priori* to the study) in independent sample sets does not generate anti-conservative results.

Considering the extraordinary significance level and effect size reported in their hypothesis generation sample set, the power to detect a similar effect even in the relatively modest sample sizes explored here is extremely high if the reported effect sizes were genuine or modestly over-estimated. Indeed, Lesnick *et al.* estimated that fewer than 100 subjects would have been required to show significance with the odds ratios they observed (see online Annotations and Discussions related to the Lesnick *et al.* publication [17]). Thus, it seems clear that factors other than variation due to random sampling are contributing to the lack of replication, particularly considering the similar demographics of the Celera sample set we have used in this study. Many factors may contribute to the negative result in our sample sets, and one important contributing factor may be that the SNP selection procedure implemented by Lesnick *et al.* provides an overly optimistic fit to the dataset on which the model was developed. In this regard, it is worth noting that most of the 23 markers showed highly significant individual association with PD risk and conferred large effect sizes in the Lesnick *et al.* dataset. For example, 19 markers had reported OR>2.0 and 13 had OR>5.0, when additive, dominant, or recessive models were considered. Such large single marker effects have rarely been observed in common complex diseases and were not replicated by the individual marker analysis in our sample sets. Lesnick *et al.* employed a bootstrap procedure to compare the significance of their axon guidance pathway SNP models to that of randomly selected SNP models and found their models to be significantly better than the randomly chosen models. However, the hypothesis tested by this procedure is not the only hypothesis of interest.

Our simulation study shows that SNP selection methods similar to those of Lesnick *et al.* can generate models with overly optimistic risk estimates and *P*-values. Although our model selection scheme did not exactly replicate the Lesnick scheme, we believe it is representative of their method. Differences in our methods included 1) the handling of missing genotype data: we removed SNPs with greater than 5% missing data and imputed missing genotypes for SNPs with ≤5% missing data, 2) selection of SNPs for final models: we performed stepwise logistic regression but did not refine with subsequent backward elimination, 3) we did not individually add SNPs back into the candidate model using stepwise selection, and 4) we did not evaluate pair-wise interactions for inclusion in our final models. Lesnick *et al.* reported that 183 of the total 1,460 axon guidance SNPs were individually associated with PD susceptibility. From these 183 SNPs they produced a final model containing 23 SNPs and 10 pair-wise interactions with an overall model *P*-value of 4.64×10^−38^. Similarly, our simulations selected a range of 82 to 174 SNPs associated with PD susceptibility from a range of 1,390 to 1,410 SNPs in randomly selected genes. Likewise, our simulated models selected a range of 14 to 58 SNPs from the 82 to 174 individually associated SNPs with overall model *P*-values ranging from 4.90×10^−64^ to 3.13×10^−23^. Apparently, our numbers of selected SNPs and associated *P*-values from the models generated from the observed but randomly selected genes as well as the permuted models are similar to those obtained by Lesnick *et al.* This suggests that their final model likely suffers from overfitting and thus should be compared to a null distribution generated by a process similar to those we have generated here to properly account for the model selection process and to provide a clear context in which to interpret the risk estimates and *P*-values of the model.

In summary, we were unable to replicate the reported finding that a putative genetic model composed of SNP variants in genes of the axon guidance pathway is a strong predictor of PD risk. We have not attempted to determine whether such models are correlated with disease-free survival or age at disease onset. Our result suggests that other genetic markers and models need to be tested to determine whether the axon guidance pathway is critically involved in PD etiology. In addition, as with reported associations of single genetic variants, our result underscores the necessity of replication for multi-marker combinations in genetic association studies [26]. We anticipate that such pathway-based or multi-gene analyses will become more common following the current wave of GWA studies [27], thus it is recommended that a vigorous replication be carried out before any disease association is proposed.

## Materials and Methods

### Study samples

Two late-onset Parkinson case-control sample sets, with a total of 525 cases and 518 controls, were used in this study (Table 1). The Celera sample set was constructed from PD cases and matched population/convenience controls that are available through the NINDS Human Genetics Resource at the Coriell Institute (http://locus.umdnj.edu/ninds). Cases and controls were matched by age and gender, where 272 pairs have identical gender and age at sampling; 39 other pairs have identical gender but age at sampling is within a 3-year interval, with most cases younger than controls. The Thessaly sample set was obtained from an outpatient clinic for movement disorders in the Larissa University Hospital in Central Greece [28]. The cases were matched to normal subjects living in the same geographical area as the cases. All cases and controls are white in both sample sets and were collected with informed consent from the individuals. Cases met UK Brain Bank criteria for idiopathic PD [29] and controls were neurologically normal. None of the cases in the two sample sets carry the Gly2019Ser mutation in *LRRK2* [30]. In addition, no significant population stratification was observed in the NINDS sample set [31]. Detailed demographics are provided in Table 1.

### Markers, assays and genotyping

The markers tested in this study include the 23 SNPs in Table 1 of Lesnick *et al.* [16] that were reported to predict PD susceptibility. Genotyping assays were developed and validated in-house using the method of allele-specific real time PCR [32]. DNA samples were individually genotyped in the Celera high-throughput genotyping lab. Genotypes were assigned by an automated algorithm, and were subjected to manual inspection by an individual who had no access to the study's phenotype information. To aid in quality control, HWE testing was carried out for each marker in the two sample sets individually. Our overall genotyping accuracy was consistently found to be >99% [33].

### Statistical analysis

HWE was evaluated using an exact test as described by Weir (“Genetic Data Analysis II”, Sinauer Associates, Sunderland MA, 1996, 2^nd^ edition). Allelic association of individual markers with disease status was evaluated using the χ^2^ test. In order to test Lesnick's model to predict PD susceptibility, we first coded each of the 23 SNPs such that the alternate allele as specified in Table S1 of Lesnick *et al.* was modeled in the log additive, dominant, or recessive coding. Next, for each subject in the dataset, the natural log of the odds ratios reported in Table 1 of Lesnick *et al.* was multiplied by the appropriately coded SNP(s) in order to represent the main and interaction effects of the model. A score for each subject was then calculated as the sum of the specified main and interaction effects. We then tested whether the score was associated with PD susceptibility in a logistic regression model. For each dataset, a second logistic regression model containing the identical main and interaction terms was run, but instead of fixing the coefficients specified by Lesnick *et al.* we allowed the coefficients to be estimated from the data. Finally, a backward stepwise selection procedure was run, with alpha≥0.05 as the condition for elimination from the model, in order to determine if eliminating some of the non-significant SNPs could result in a better model.

A simple Monte Carlo simulation, written in XLISP-STAT, was performed for illustration purposes of the overfitting present when multiple testing is not accounted for. 198k SNPs were generated under the null hypothesis of independence between case-control status and genotype count as a source of the randomly-selected SNP (although 198k SNPs were generated, really only one SNP was necessary as these were randomly-selected). The selected SNPs were derived from the most significant SNP from 1,400 SNPs also generated under the null model. Allele frequency spectrum was derived from a beta distribution similar to those studied in neutral models of population genetics. The distribution was then truncated (frequencies were bounded by 2% and 98%) to model the filtering of SNPs that often occurs on the basis of frequency for GWA panels. HWE was explicitly used. The subsequent generation of genotype counts was identical for cases and controls. A 2-df likelihood ratio test of genotypes between cases and controls was used to calculate association *P*-values. The simulation was iterated 200 times and a randomly-selected SNP and the most significant SNP were saved after each iteration.

The publicly available data from Fung *et al.* [8] was used to simulate a null distribution which accounts for the potential overfitting incurred when a model is built using a set of SNPs selected from among a large set of SNPs. We constructed 500 models using SNPs selected from randomly chosen genes (Observed Models) and following a selection scheme similar to that of Lesnick *et al.* and an additional 500 models by permuting the disease status of the study participants prior to simulating the selection scheme (Permuted Models). To begin, 500 datasets were constructed by serially randomly selecting genes from the Fung *et al.* data and including all SNPs from the selected genes until there were no less than 1,390 SNPs and no more than 1,410 SNPs in the datasets. From these 500 datasets, 500 parallel datasets were constructed by permuting the disease status of the study participants. SNPs with >5% missing genotype data were eliminated from consideration in the model selection process. Missing genotypes for SNPs with ≤5% missing data were imputed using Hardy-Weinberg genotype frequencies derived from empirical allele frequencies stratified by disease status. For each dataset, we followed Steps 1, 2, and 3 of Lesnick's model selection scheme to select individual SNPs to be included for subsequent selection into final models. Specifically, genotypes for each of the selected SNPs were coded as genetically log-additive, Mendelian dominant, and Mendelian recessive. Logistic regression was employed to select individual SNPs associated with PD for each genetic coding. Wald χ^2^ statistics were recorded for each SNP and those with *P*-values>0.05 were removed from further consideration. The smallest *P*-value of the three codings for each SNP was identified and those SNPs with *P*-values≤0.05 were included in a stepwise logistic regression procedure with the individually associated SNPs from above used as the set of variables considered for inclusion in the model. Likelihood ratio tests were performed on the overall final stepwise models and *P*-values were recorded for each of the 500 observed models and the 500 permuted models.
